# Limited Mouth Opening Secondary to Diffuse Systemic Sclerosis

**DOI:** 10.1155/2013/937487

**Published:** 2013-12-18

**Authors:** Tomoko Wada, Saravanan Ram

**Affiliations:** ^1^Orofacial Pain and Oral Medicine Center, Ostrow School of Dentistry of USC, Los Angeles, CA 90089, USA; ^2^Orofacial Pain and Oral Medicine Residency Program, Orofacial Pain and Oral Medicine Center, Ostrow School of Dentistry of USC, Los Angeles, CA 90089, USA

## Abstract

Systemic sclerosis (SSc) is a relatively rare condition with an immunologically mediated pathogenesis. For reasons that are not clearly understood, dense collagen is deposited in the connective tissues of the body in extraordinary amounts. Although its dramatic effects are seen in association with the skin, the disease is often quite serious with visceral organ involvement. We describe a case of limited mouth opening secondary to diffuse SSc, improvement in mouth opening with passive jaw stretch exercises, and the challenges involved in performing dental procedures for such patients.

## 1. Introduction

Systemic sclerosis (SSc) or scleroderma is an incurable and potentially life-threatening systemic autoimmune connective tissue disease of unknown etiology and multifactorial pathogenesis characterized by cutaneous and visceral fibrosis, microvascular obliteration, and highly specific serum autoantibodies to nuclear autoantigens [1, 2]. SSc is divided into diffuse cutaneous, and limited cutaneous forms based on the extent of skin involvement. Various genetic, infectious and environmental factors have been implicated in the etiopathogenesis of SSc [3].

Orofacial tissue involvement is a typical feature in patients suffering from SSc; the subject's face becomes expressionless, the cutaneous furrows disappear, and the nose becomes sharp. The alterations in facial form also include decreased mouth opening (microstomia). This may interfere considerably with eating, speaking, oral hygiene measures, and dental treatment, thus deteriorating the quality of life of these subjects [4, 5]. This paper describes a case of limited mouth opening secondary to diffuse SSc, improvement in mouth opening with passive jaw stretch exercises, and the challenges involved in performing dental procedures for such patients.

## 2. Case Report

**Figure 1:**
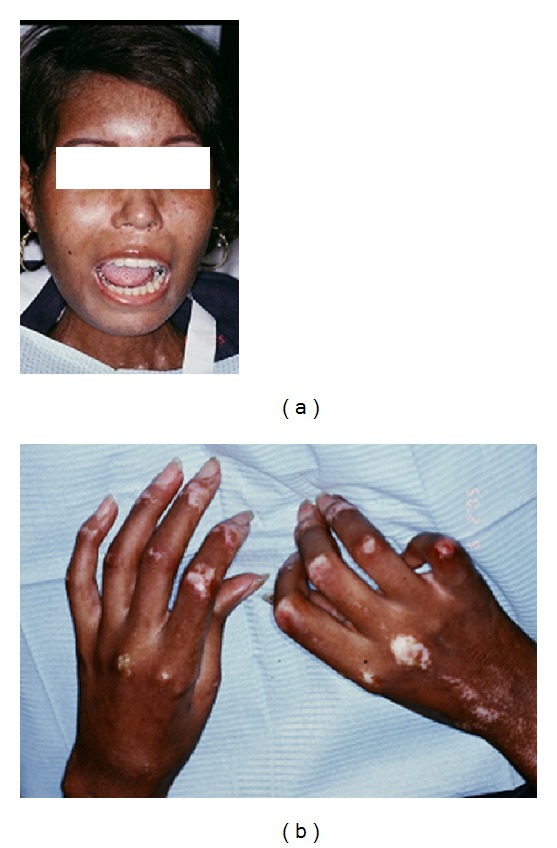
(a) The involvement of the facial skin with abnormal collagen deposition produces a mask-like face. Note the hypopigmentation, telangiectasias, loss of alae of the nose, and taut skin around the neck. (b) Sclerodactyly with hypopigmentation, telangiectasias, and flexion contractures of the fingers.

A 22-year-old African American woman reported to Orofacial Pain and Oral Medicine Center at the Ostrow School of Dentistry, University of Southern California, with a complaint of limited mouth opening. The patient had been referred by her general dentist for evaluation and management of her limited mouth opening. Examination of the patient revealed a narrow mask-like face, with taut facial and cervical skin (Figure 1(a)). Her blood test was positive for Anti-Scl70 (topoisomerase I) antibodies typical of diffuse SSc. The patient's maximum mouth opening was 10 mm with a hard end feel on passive stretch. Examination of the hands revealed deformed fingers with taut skin indicative of sclerodactyly (Figure 1(b)). Her medical history was significant for diffuse cutaneous SSc since the age of 18, migraines, depression, decreased gastrointestinal motility leading to gastro-esophageal reflux disease, and mild mitral regurgitation. A panoramic radiograph was taken which showed normal condyles and normal mandibular rami. Bite wing radiographs were taken which showed no evidence of dental caries or periodontal disease. We were unable to perform a full mouth radiographic series due to the limited mouth opening.

**Figure 2:**
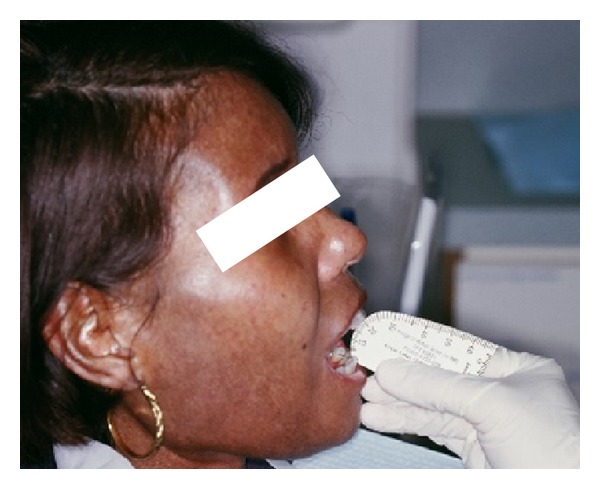
Improved mouth opening of 20 mm following passive stretch exercises for the jaw.

The patient was advised to do passive jaw stretch exercises using stacks of tongue blades six times per session, six sessions a day. The patient was advised to increase the number of tongue blades used if she felt an improvement in the mouth opening. The patient diligently performed the passive jaw stretch exercises and her mouth opening improved to 20 mm during her 6-week follow-up visit (Figure 2). Upon reevaluation of the patient at six-month and one year intervals, there were no new cavities or periodontal disease and the mouth opening was 20 mm.

## 3. Discussion 

Recent incidence and prevalence data show that SSc occurs more commonly in women than in men [6], particularly in the age range 45–64 years; the minimum estimated values of incidence and prevalence are 20/million per year and 1,500/million, respectively [7, 8]. In terms of race/ethnicity, African Americans have been reported to have earlier onset and more severe disease [6] as was seen in our case.

Although the pathogenesis of SSc has not been fully elucidated yet, the fibrosis is characterized by the excessive accumulation of extracellular matrix proteins in the skin and viscera with vascular injury and immunological abnormalities. Collagen deposition in connective tissue leads to fibrosis and progressive limitation of mouth opening [3]. The skin develops a diffuse, hard texture, and its surface is usually smooth. Limited mouth opening is a frequent finding in patients with SSc [5]. Bilateral or unilateral commissurotomy has been described as a surgical means of correcting the limited mouth opening in SSc patients [9, 10]. A low-cost nonsurgical alternative to improve limited mouth opening involves the use of two stacks of tongue blades inserted between the posterior teeth to increase the size of mandibular opening. Patient compliance and perseverance are critical factors for successful treatment outcome [11]. Pizzo et al. [5] showed an average increase in mouth opening of 10.7 ± 2.06 mm with jaw stretch exercises. In our case, the mouth opening improved by 10 mm with passive jaw stretch exercises for six weeks.

From a dental perspective, problems may develop for patients who wear prostheses due to the limited mouth opening and inelasticity of the mouth [12]. Patients may also have problems with maintaining good oral hygiene, and they have decreased ability to manipulate a tooth brush as a result of sclerotic changes in the fingers and hands, as was seen in our case. Adapted equipment may make it easier for a patient with decreased dexterity and range of motion to brush and floss one's teeth [13].

Xerostomia is frequently seen with the possibility of concurrent secondary Sjogren's syndrome [14]. Dental radiographs may show diffuse widening of the periodontal ligament space throughout the dentition. Varying degrees of resorption of the posterior ramus of the mandible, the coronoid process, the chin, and the condyle may be detected on panoramic radiographs affecting approximately 10–20% of patients [15]. In our case, there was no subjective complaint or objective evidence of xerostomia. The radiographs showed no evidence of periodontal ligament space widening. The mandibular rami, condyles, and coronoid process were within normal limits with no evidence of resorption.

Dentists should be aware of the challenges associated with performing routine intraoral examination, periapical radiographs, and dental treatment for SSc patients with limited mouth opening. Passive jaw stretch exercises using tongue blades are modestly effective in improving the limited mouth opening in SSc patients.
